# Fertility-Sparing Treatment in Young Patients With Grade 2 Presumed Stage IA Endometrioid Endometrial Adenocarcinoma

**DOI:** 10.3389/fonc.2020.01437

**Published:** 2020-08-25

**Authors:** Mei Yu, Yao Wang, Zhen Yuan, Xuan Zong, Xiao Huo, Dong-yan Cao, Jia-xin Yang, Keng Shen

**Affiliations:** Department of Obstetrics and Gynecology, Peking Union Medical College Hospital, Chinese Academy of Medical Science and Peking Union Medical College, Beijing, China

**Keywords:** fertility-sparing treatment, grade 2 endometrioid endometrial cancer, hormonal therapy, LNG-IUS, GnRH-a

## Abstract

**Purpose:** To investigate the efficacy of fertility-sparing treatment for young women with grade 2 presumed stage IA endometrioid endometrial adenocarcinoma (EEA).

**Methods:** We performed a retrospectively review of eight patients affected by grade 2 presumed stage IA endometrioid endometrial adenocarcinoma who underwent fertility-sparing treatment in the Peking Union Medical College Hospital between 2011 and 2018.

**Results:** The median age of patients was 26 years (range, 22–35 years). Complete response (CR) was found in seven of the eight cases. The median time to response was 3 months (range, 3–9 months). Among patients who achieved CR, three had recurrence and were treated with second-line fertility-sparing therapy. Two of the three recurrent patients achieved CR, and one patient subsequently conceived. Pregnancies and successful deliveries were achieved in two of four patients. The average follow-up period was 31 months (range, 21–77 months).

**Conclusions:** Fertility-sparing therapy is a feasible treatment option in patients with presumed stage IA, grade 2 endometrial cancer. Although our results are encouraging, they are based on very limited numbers, and patients should be informed the risk of tumor progression during treatment. Further evaluations are still required before recommending fertility-sparing therapy to endometrial cancer patients with more advanced disease in routine practice.

## Introduction

Endometrial carcinoma (EC) is the most common gynecological malignancy in the world. It is typically diagnosed in post-menopausal women. The standard treatment for EC consists of hysterectomy and bilateral salpingo-oophorectomy with or without pelvic and para-aortic lymph node assessment (1). This treatment precludes future fertility and may thus be undesirable for women wishing to preserve their fertility.

Approximately 3–14% of endometrial cancer cases are diagnosed in women equal to or under 40 years of age who want to preserve their fertility (2, 3). The conservative treatment for young women with G1 EC and atypical hyperplasia (AH) who desire to conceive has been proved to be a safe and feasible therapy (4–8). Nevertheless, for women affected by grade 2 disease, there is limited literature regarding this issue, and the data correspondingly are limited. There has been no prospective trial on the effectiveness of fertility-sparing treatment in patients with grade 2 or more advanced disease. Therefore, the aim of this study was to investigate the efficacy of conservative management in patients with early-stage grade 2 endometrial cancer who desired to preserve their fertility.

## Methods

The medical records of patients with grade 2 presumed stage IA endometrioid endometrial adenocarcinoma (EEA) who underwent fertility-sparing treatment at the Peking Union Medical College Hospital between 2011 and 2018 were retrospectively reviewed. The Institutional Review Board (IRB) of “the Ethics Committee of Peking Union Medical College Hospital” approved the use of patients' clinical data for this retrospective study. Patients must be informed that data about fertility-sparing treatment are not so robust to draw firm conclusion due to the limitation in sample size of available studies, and, most importantly, there is a risk of disease progression during treatment or after the initial response. All patients were informed that hormone therapy is not the standard treatment and requires serial dilation and curettage (D&C) or hysteroscopy during treatment period. Inclusion criteria were fertile age (40 years or younger), desire to preserve fertility, and histologically proven grade 2 EEA, tumor presumed to be International Federation of Gynecology and Obstetrics (FIGO) stage IA limited to the endometrium. The diagnosis was confirmed by endometrial biopsy under hysteroscopy. All of the histological slides were reviewed by at least two pathologists specializing in gynecological oncology. All patients were evaluated by pelvic examination, ultrasound scan/abdominal, and pelvic computed tomography/pelvic magnetic resonance imaging at baseline to confirm tumor limited into the endometrium and no evidence of lymph node involvement or extrauterine metastasis.

Patients were scheduled to receive medroxyprogesterone acetate (MPA) 500 mg qd or megestrol acetate (MA) 160 mg bid orally on a daily basis or the combination of intramuscular injections of 3.75 mg gonadotropin-releasing hormone agonist (GnRHa) every 4 weeks plus levonorgestrel-intrauterine system (LNG-IUS) inserted. Response was assessed histologically every 3 months after the start of treatment. Complete blood counts and biochemistry panels along with renal function and liver profiles were performed every month, and serum CA125 and pelvic ultrasound were carried out every 3 months. Endometrial tissue sampling for diagnosis should be carried out by hysteroscopy and endometrial curettage or D&C. All histology was reported by two expert pathologists specialized in gynecological oncology. Pathological response to MPA treatment was categorized as complete response (CR), partial response (PR), stable disease (SD), and progressive disease (PD). CR was defined as the absence of evidence of hyperplasia or carcinoma. PR was defined as histological regression or endometrial decidual change. SD was defined as the persistence of EC or AH. PD was defined as progression to a lesion of higher grade or clinically progressive disease including myometrial invasion, extrauterine disease, or lymph node metastasis (9). Evaluation was performed every 3 months via hysteroscopy; patients with PR or SD continued the same treatment for an additional 3 months or change the dose, whereas those with PD were immediately proposed to receive surgery. Patients with CR who desire to become pregnant were encouraged to conceive or referred to *in vitro* fertilization (IVF) immediately. Those with CR who opted to postpone pregnancy were prescribed oral contraceptives/progestin or LNG-IUS to prevent cancer recurrence.

## Results

Between 2011 and 2018, there were eight patients included in this study (Table 1). The median age of patients was 26 years (range, 22–35 years). All patients desired to preserve fertility and accepted fertility-sparing treatment. Patients in the study were treated with MA or MPA or the combination of GnRHa plus LNG-IUS initially. Patients' treatment regimen, therapeutic response, recurrence, and oncologic and fertility outcomes are presented in Table 2.

**Table 1:** The clinicopathologic characteristics of the study participants.

**No**.	**Age**	**Complications**			**CA125**	**Recurrent pathology**
		**Infertility**	**PCOS**	**Obesity (BMI, kg/m^**2**^)**	**(U/ml)**	
1	31	No	Yes	No (27.9)	46.8	–
2	23	No	Yes	Yes (32.7)	37.5	AH2-3
3	31	No	No	No (22.0)	56.7	–
4	27	Yes	No	Yes (31.2)	27.8	G2-3 EA
5	26	Yes	Yes	Yes (37.6)	27.5	–
6	35	No	No	Yes (30.5)	32.0	–
6	26	Yes	Yes	No (29.4)	40.7	AH 3
8	22	No	Yes	No (28.6)	30.5	–

*Obesity, BMI ≥ 30 kg/m^2^; EA, endometrioid adenocarcinoma; AH, atypical hyperplasia; G, grade; PCOS, polycystic ovary syndrome*.

**Table 2:** Clinical characteristics and oncologic and fertility outcomes of eight patients with G2EA.

**Patients**	**Age**	**Initial disease**				**Recurrent disease**					**Pregnancy**	**Status, Follow-up (months)**
		**Initial diagnosis**	**Initial treatment**	**CR**	**Time to CR (months)**	**Time to recurrence (months)**	**Recurrent diagnosis**	**Repeated treatment**	**Response to retreatment (months)**	**Hysterectomy (pathology)**		
1	31	G1-2 EA	MPA 500 mg/day	N	/	/	/	/	/	Y (EA, IIIC1)	/	NED (21)
2	23	G1-2 EA	MA 160 mg bid → GnRHa every 4 weeks + LNG-IUS	Y	9	Y (17)	AH2-3	GnRHa every 4 weeks + LNG-IUS	CR (3)	N	Suggest IVF	NED (28)
3	31	G2 EA	MA 160 mg bid	Y	3	N	/	/	/	N	NFTD	NED (31)
4	27	G1-2 EA	MA 160 mg bid	Y	3	Y (24)	G2-3 EA	MA 160 mg bid	NC (4)	Y (EA, IIIC1)	/	NED (30)
5	26	G1-2 EA	GnRHa every 4 weeks + aromatase inhibitor + LNG-IUS	Y	3	N	/	/	/	N	Postpone pregnancy	NED (33)
6	35	G1-2 EA	MPA 500 mg/day	Y	3	N	/	/	/	Y (EA, IA)	/	NED (31)
7	26	G1 EA, focal lesions ofG2	MA 160 mg bid	Y	6	Y (36)	AH3	MA 160 mg bid	CR (6)	N	NFTD	NED (39)
8	22	G1-2 EA	MPA 500 mg/day	Y	6	N	/	/	/	N	Unmarried	NED (77)

*MPA, medroxyprogesterone acetate; MA, megestrol acetate; IVF, in-vitro fertilization; GnRHa, gonadotropin-releasing hormone agonist; LNG-IUS, levonorgestrel-intrauterine system; EA, endometrioid adenocarcinoma; AH, atypical hyperplasia; CR, complete response; NC, no change; NED, no evidence of disease; NFTD, normal full-term delivery; G, grade; Y, yes; N, no*.

Of the eight patients, seven (87.5%) achieved CR. The median time to CR was 3 months (range, 3–9 months). One patient (patient 2) changed therapeutic regimen in initial treatment due to no response to initial 3 months of progestin therapy. The only patient (patient 1) who failed to respond to initial 7 months of hormone treatment finally underwent hysterectomy with surgical staging. The post-operatively histopathological result showed FIGO stage IIIC1. This patient, who was treated with chemotherapy and radiotherapy after surgery, was alive and free of disease at last follow-up.

Three of the seven who achieved CR (patients 2, 4, and 7) developed recurrent disease. The time to recurrence was 17, 24, and 36 months, respectively. After recurrence with G2-3 endometrial carcinoma, patient 4 who insisted on receiving 4 months of MA failed to respond. She underwent hysterectomy with surgical staging. The final pathology of the uterus was FIGO stage IIIC1 grade 3 EC, and she received chemotherapy post-operatively and was free of disease at last follow-up. The remaining two (patients 2 and 7) who still desired fertility were treated with second round of fertility-sparing management and got CR again. Patient 2 with the recurrent pathology of AH2-3 were treated with the combination of intramuscular injections of GnRHa every 4 weeks combined with LNG-IUS. She achieved CR again after 3 months and is undergoing *in vitro* fertilization and embryo transfer (IVF-ET). Patient 7 delivered successfully after the first recurrence and relapsed again with pathology of AH3. She achieved CR at 6 months after a third round progestin treatment using MA 160 mg bid after the second recurrence. Among the eight patients, one patient (patient 6) underwent hysterectomy according to the patient's decision even though she showed CR.

Of the seven patients with complete remission, three patients desired to conceive immediately, and two patients had pregnancies and successfully delivered during the follow-up period.

The remaining patients (patient 5, 6, and 8) are all alive and free of disease. The median follow-up of the eight patients was 31 months (range, 21–77 months), with no disease-related death and no major adverse effects related to high-dose progestin.

## Discussion

The incidence of endometrial cancer is increasing with lower average onset age, and most patients have not yet completed the reproductive function. Therefore, fertility-sparing treatment has received much attention during recent years. Since progesterone was first reported to be used in the fertility-sparing treatment of early endometrial cancer, researchers continue to validate that conservative treatment for young women with G1 EC and AH who desire to conceive is a safe and feasible therapy. Previous literature shows that oral progestin therapy can achieve CR rates ranging between 67 and 80% (4–8). Our recent meta-analysis found that among 445 grade 1 stage IA EC patients who administered oral progestin, the remission rate was 82.4% (10). The pregnancy rate was 28.8%, and the recurrence rate was 25% (10). The differentiation degree of EC is the most important indicator to predict stage and response to progestin treatment (11). Duska et al. (12) reviewed women with EC under the age of 40, and the results showed that only grade 1 EC could predict stage I disease among them. In addition, Thigpen et al. (13) demonstrated that in advanced or recurrent EC, the response rate to MPA was 37% for grade 1 tumors compared with 23% for grade 2 and 9% for grade 3 tumors. Looking through the current literature, there are few reported cases of progestin-based fertility-sparing therapy in young women with intramucosal G2-3 EC. According to the Mayo Clinic's experience, the risk of lymphatic spread in low-risk women (patients preoperatively diagnosis with G1 or G2, endometrioid histological type, and tumor diameter B2.0 cm) is <1% (14). Therefore, fertility-sparing treatment could be expanded to stage IA type I and G2 EC since they are generally considered to be “low-risk” population.

To date, there are 15 articles regarding the fertility-sparing treatment of stage IA, G2 EC in young patients (15–29) (Table 3). Taking together the data from the papers, 38 (67.9%) of 56 patients achieved CR, and 16 (42.1%) of those 38 CR cases later showed recurrent disease. Park et al. (25) conducted a multicenter retrospective cohort study to estimate the oncologic and pregnancy outcomes after oral MPA or MA treatment of patients with grade 2 or 3 stage IA EC. Among patients without myometrial invasion, the CR rate and the recurrence rate were 76.5% (13/17) and 23.1% (3/13), respectively. Our study demonstrated that the CR rate for G2 EC was 87.5% (7/8) with a median time to CR of 3 months, and the recurrence rate after complete remission was 42.8% (3/7). These results are comparable to those of patients with stage IA, grade 1 differentiation without myometrial invasion. Due to the limitations of retrospective study design and the small sample size, more prospective large-scale studies will have to await further.

**Table 3:** Outcomes of conservative treatment in patients with grade 2 stage IA EC.

**Authors**	**Year**	**Number of cases/patient number**	**Progestin therapy**	**Response**	**Response month**	**Recurrence**	**Hysterectomy**	**Pregnancy**	**Follow-up time (months)**	**Status**
Bokhman et al. (15)	1985	8	Hydroxyprogesterone 500 mg/day for at least 3 months	–	–	–	–	–	–	Regression
Kim et al. (16)	1997	Patient1	MA 160 mg/day	NC	–	–	–	–	8	NED
Sardi et al. (17)	1998	Patient1	MPA 50 mg/day	NC	–	–	Y (minimal myometrial invasion)	N	20	NED
Zuckerman et al. (18)	1998	Patient1	MPA(not reported)	CR	2	N	Y (no lesion)	Y (twin IVF)	Not reported	NED
Imai et al. (19)	2001	Patient1 Patient2	MPA 600 mg/day MPA 600 mg/day	CR NC	9 –	Y (7) –	N Y (focal adenocarcinoma)	N N	Lost to follow 47	– NED
Kaku et al. (20)	2001	Patient1 Patient2	MPA 800 mg/day MPA 600 mg/day	CR NC	4 –	N –	N Y (EA, G2, MI+)	NFTD N	19 22	NED NED
Gotlieb et al. (21)	2003	Patient1	MPA 200 mg for 14 days, every 4 weeks	CR	3	Y (40)	Y (EA, G1)	NFTD	94	NED
Han et a. (22)	2009	Patient1 Patient2	MPA 500 mg/day MA 80 mg/day	CR CR	3 3	N N	N N		52 42	NED NED
Koskas et al. (23)	2011	Patient1 Patient2 Patient3	NES 20 mg/day MA 160 mg/day NG 5 mg/day	CR CR CR	3 6 3	Y (3) N Y (36)	Refused N Y (EA,G1)	N Y (twin) N	12 24 60	EA, G1 NED NED
Brown et al. (24)	2012	Patient1	LNG-IUS	CR	3	N	N	N	13	NED
Park et al. (25)	2013	23	MA 160 mg/day or MPA 500 mg/day	17 (73.9%)	17 (9–51)	8 (47.1%)		5 (45%)	49 (22–95)	NED
Rossetti et al. (26)	2014	Patient1 Patient2	MA 160 mg/day MA 160 mg/day	CR CR	3 3	N N	Y N		52 36	NEDNED
Hwang et al. (27)	2017	Patient1 Patient2 Patient3 Patient4 Patient5	combined oral MPA (500 mg/day)/LNG-IUS	CR CR PR CR PR	11 ± 6.2 (6–18)	1 (20%)	N N N N Y (EA, no MI)	Yes(missed Abortion) N N N N	59 19 10 55 69	NED NED NED NED NED
Pal et al. (28)	2018	8	LNG-IUS	3 (37.5%)	Not reported	Not reported	Not reported	Not reported	Not reported	Not reported
Maggiore (29)	2019	4	LNG-IUS	3 (75%)	4 ± 0	3 (100%)	Y	N	115.5 ± 2.6	Not Reported
Total				38/56 (67.9%)		16/38 (42.1%)				

*EA, endometrioid adenocarcinoma; G, grade; IVF, in vitro fertilization; MA, megestrol acetate; MI, myometrial invasion; NED, no evidence of disease; NC, no change; NES, norethisterone; NFTD, normal full-term delivery; NG, nomegestrol; LNG-IUS, levonorgestrel-intrauterine system; Y, yes; N, no*.

Conservative treatment of endometrial adenocarcinoma traditionally involved oral progestin therapy; more recently, many reports have introduced LNG-IUS alone or combined with GnRHa or oral progestin as an alternative to oral systemic progestin alone for the treatment of women with EC and AH (24, 27–30). Brown et al. (24) in 2012 reported a case of an 18-year-old girl with grade 2 endometrioid adenocarcinoma, treated with LNG-IUS successfully. Hwang et al. (27) evaluated the efficacy of combined MPA/LNG-IUS treatment for grade 2 stage IA EC; the CR rate was 60% (3/5), and one patient (13.3%) who relapsed after 14 months achieving CR received combined therapy and achieved CR by 6 months again. A prospective observational study showed that for grade 1 EC, combined oral MPA/LNG-IUS treatment was more effective than oral progestin alone (31). The above findings suggested that, as a fertility-sparing option, compared with oral progestin alone, combined therapy may produce more favorable outcomes for grade 2 EC. In our study, two patients achieved CR by using GnRHa combined with LNG-IUS treatment, and one of them changed regimen from MA to the combination of GnRHa and LNG-IUS due to ineffective treatment. Our result suggested that the combination of GnRHa with LNG-IUS represents an effective therapeutic approach for patients with G2 EC.

Most of the patients who underwent hysterectomy after conservative treatment failure were confirmed to be early clinical stage, well-differentiated endometrioid adenocarcinoma, indicating the disease was less likely to progress during hormone therapy. However, in our study, one patient who failed to respond to 7 months of hormone treatment underwent staging surgery, and the final pathology suggested stage IIIC1 EC. According to the European Society of Gynecological Oncology recommendation, we should consider it unsuitable for patients with persistent or progressive disease after 6 months of progestin treatment to continue conservative treatment (32). As for this patient, the disease was stable in the first 6 months treatment, and she insist on trying another cycle of treatment. Unfortunately, during the seventh month of treatment, she experienced disease progression. Another patient with recurrence receiving 4 months of MA and suffering no change of lesions underwent definitive surgery with a diagnosis of stage IIIC1 EC. Although these two patients are alive and free of disease at last follow-up, we should be aware of the risk of disease progression during fertility-sparing treatment. It should be considered that poorly differentiated EC patients who received fertility-sparing treatment be counseled on the need for close follow-up, as well as the dangers of delaying hysterectomy.

There are few papers focusing on the pregnancy outcome of fertility-sparing treatment in patients with intramucosal G2-3 EC (18, 20, 21, 23, 25, 27). In the present series, although only 69.2% of patients who achieve CR attempted to conceive during the study, the pregnancy and live birth rates were 37% and 33%, respectively. While in our study, the overall live birth rate was 66.7%. It should be recommended that patients achieving CR should consult a reproductive endocrinologist to maximize the possibility of a live birth and minimize the time between diagnosis and definitive EC treatment.

## Limitations

Although our study is a single-center retrospective study with a limited number of cases, we believe it can provide useful clinical data to support the feasibility of conservative treatment in patients with stage IA G2 EC. Meanwhile, it is necessary to conduct a regional or international randomized clinical trial that focused only on fertility-sparing treatment in patients with stage IA G2 EC in order to clarify the oncologic and fertility outcomes. The fertility-preserving treatment for early EC should be performed on the basis of the following requirements: first of all, a good prognosis after fertility-sparing therapy (exceeding 95% in 5-year survival rate); second, in terms of overall survival rate, fertility-sparing therapy should be not inferior to standard surgical treatment. At present, G2 intramucosal endometrial cancer is a low-risk type that does not require adjuvant treatment after primary surgery. However, the long-term prognosis is not clear because of the limited number of cases. The follow-up time of this center is limited, and long-term follow-up of these patients will also be performed to verify long-term survival.

## Conclusions

Based on our own encouraging results, fertility-sparing treatment can be a feasible and effective option for patients with grade 2 presumed stage IA EC without myometrial invasion who desire to preserve their fertility. However, conservative management in this population should still be considered with caution, and patients need to be monitored more closely so that fertility-sparing treatment can be terminated in time when patients failed to respond to treatment.

## Data Availability Statement

The datasets generated for this study are available on request to the corresponding author.

## Ethics Statement

This retrospective study had obtained approval of the Institutional Ethical Committee of Peking Union Medical College Hospital. All patients had signed an informed consent.

## Author Contributions

MY, JY, DC, and KS: conceived and designed the study. MY, JY, DC, and XH: data acquisition. YW, MY, ZY, and XZ: analyzed the data. MY: wrote the original draft. YW, MY, JY, DC, and KS: wrote, reviewed, and edit. All authors contributed to the article and approved the submitted version.

## Conflict of Interest

The authors declare that the research was conducted in the absence of any commercial or financial relationships that could be construed as a potential conflict of interest.
